# Association of Preoperative Fasting Strategy with Postoperative Inflammatory–Nutritional Trajectories and Clinical Outcomes Following Hepatobiliary Surgery: A Retrospective Cohort Study

**DOI:** 10.3390/nu18172922

**Published:** 2026-09-07

**Authors:** Hyun-Jung Kwon, Suryeon Lee, Yong-Hee Park, Sujung Park, Sung Moon Jeong, Dae Wook Hwang, Seihee Min

**Affiliations:** 1 Department of Anesthesiology and Pain Medicine, Asan Medical Center, University of Ulsan College of Medicine, Seoul 05505, Republic of Korea; 2 Department of Surgery, Division of Hepatobiliary and Pancreatic Surgery, Asan Medical Center, University of Ulsan College of Medicine, Seoul 05505, Republic of Korea

**Keywords:** fasting, preoperative care, nutrition assessment, postoperative complications

## Abstract

**Background/Objectives**: Major hepatobiliary surgery elicits substantial inflammatory and catabolic stress, which may be aggravated by prolonged preoperative fasting. We evaluated the association of preoperative fasting strategy with postoperative inflammatory–nutritional trajectories and clinical outcomes after elective hepatobiliary surgery. **Methods**: This retrospective cohort study included 1409 adults who underwent elective hepatobiliary surgery between January 2020 and December 2025. Patients were classified according to the interval from last caloric intake to anesthesia induction as <6 h or ≥6 h. All patients in the <6 h group were managed under an Enhanced Recovery After Surgery (ERAS) pathway with preoperative carbohydrate loading. Postoperative trajectories of the *C*-reactive protein-to-albumin ratio (CAR), neutrophil-to-lymphocyte ratio (NLR), and prognostic nutritional index (PNI) were analyzed using linear mixed-effects models. **Results**: The <6 h group showed more favorable postoperative CAR and NLR trajectories (group × time interaction, both *p* < 0.001). Although the overall PNI trajectory also differed between groups (*p* = 0.003), between-group differences were inconsistent across individual time points. In fully adjusted models, longer fasting duration was associated with higher postoperative day (POD) 3 CAR and NLR, but not with PNI. After additional adjustment for ERAS implementation, the CAR and NLR associations were attenuated and no longer statistically significant. Clinical outcomes did not differ between groups. **Conclusions**: An ERAS-associated shortened-fasting strategy incorporating preoperative carbohydrate loading was associated with more favorable postoperative CAR and NLR trajectories. These findings reflect associations with the overall perioperative strategy rather than an independent effect of fasting duration.

## 1. Introduction

Hepatobiliary surgery is among the most invasive abdominal procedures and is associated with a pronounced systemic inflammatory response accompanied by substantial metabolic and nutritional disturbances [1]. This perioperative stress response is characterized by the release of proinflammatory cytokines, activation of the hepatic acute-phase response, increased protein catabolism, and impaired glucose homeostasis, all of which contribute to postoperative complications, prolonged hospitalization, and delayed recovery [1,2,3]. Because the severity and resolution of these responses are closely associated with postoperative recovery, reliable assessment of perioperative inflammatory and nutritional status has become increasingly important.

Among the available biomarkers, *C*-reactive protein (CRP) and serum albumin are widely used to evaluate postoperative inflammation and nutritional status [4,5]. However, each marker alone is influenced by multiple perioperative factors and may not adequately reflect the complex interaction between systemic inflammation and nutritional reserve. The CRP-to-albumin ratio (CAR), calculated from CRP and serum albumin concentrations, reflects systemic inflammation and nutritional status, and has been associated with postoperative complications and adverse outcomes in various surgical populations [6,7,8,9]. Additional biomarkers, including the neutrophil-to-lymphocyte ratio (NLR) and prognostic nutritional index (PNI), provide complementary information on systemic immune activation and nutritional reserve, and have been associated with postoperative complications and prognosis [9,10,11,12].

Optimizing preoperative fasting has become a major component of modern perioperative care. Enhanced Recovery After Surgery (ERAS) guidelines recommend minimizing unnecessary fasting and allowing preoperative carbohydrate-containing beverages in appropriately selected patients to attenuate perioperative metabolic stress and improve insulin sensitivity [13,14,15,16,17,18]. These interventions are expected to influence postoperative inflammatory and nutritional responses by reducing insulin resistance and preserving metabolic homeostasis. Nevertheless, despite their established metabolic benefits, whether shortened preoperative fasting alters the longitudinal postoperative trajectories of inflammatory–nutritional biomarkers following major hepatobiliary surgery remains incompletely understood.

In this retrospective cohort study, we investigated the association between preoperative fasting strategy, categorized according to the interval between the last caloric intake and induction of anesthesia (<6 h vs. ≥6 h), and postoperative inflammatory–nutritional trajectories after elective hepatobiliary surgery. Because preoperative carbohydrate-containing beverages were administered as part of the institutional ERAS pathway, the shortened-fasting category represented an ERAS-associated perioperative strategy incorporating carbohydrate loading rather than fasting duration in isolation. The primary outcome was the postoperative trajectory of CAR, while secondary outcomes included the trajectories of NLR and PNI, postoperative clinical outcomes, and continuous associations between fasting duration and inflammatory–nutritional biomarkers on POD 3. We hypothesized that the ERAS-associated shortened-fasting strategy would be associated with more favorable postoperative inflammatory–nutritional trajectories.

## 2. Materials and Methods

### 2.1. Study Design and Ethical Approval

This retrospective cohort study was approved by the Institutional Review Board (IRB) of Asan Medical Center (IRB No. 2026-0113; approval date: 23 January 2026; principal investigator: Seihee Min, M.D., Ph.D.). The requirement for written informed consent was waived owing to the retrospective nature of the study. This manuscript adheres to the Strengthening the Reporting of Observational Studies in Epidemiology (STROBE) guidelines.

### 2.2. Study Population

Adult patients aged ≥18 years who underwent elective hepatobiliary surgery at Asan Medical Center between January 2020 and December 2025 were retrospectively identified from the electronic medical record system. Patients were eligible if they had documented preoperative fasting information and available perioperative laboratory data for calculating inflammatory and nutritional indices. Patients with unclear or undocumented fasting information, preoperative CRP concentrations > 100 mg/L suggesting severe systemic inflammation or sepsis, preoperative serum albumin concentrations < 2.5 g/dL suggesting severe malnutrition, emergency surgery, or death during surgery or within 24 h postoperatively were excluded.

### 2.3. Preoperative Fasting Protocol and ERAS Implementation

Preoperative fasting duration was defined as the elapsed time, in minutes, from the last documented caloric intake, including solid food or a carbohydrate-containing beverage, to the induction of anesthesia. When separate records for solid food and liquids were available, the most recent caloric intake before surgery was used as the reference point.

Patients managed according to the institutional ERAS pathway were encouraged to consume preoperative oral carbohydrate-containing beverages as part of the standardized perioperative protocol. The carbohydrate regimen consisted of Encover solution (JW Pharmaceutical, Seoul, Republic of Korea; 200 mL, 1 kcal/mL, containing 31.24 g of carbohydrates), administered approximately 8–12 h before anesthesia induction, followed by Nucare NONPO (DAESANG, Seoul, Republic of Korea; 200 mL, 1 kcal/mL, containing 12.8% carbohydrates), administered approximately 2 h before anesthesia induction. Patients managed with the conventional fasting protocol did not receive preoperative carbohydrate-containing beverages. In the final analytic cohort, all 897 patients with fasting duration <6 h were managed under the ERAS pathway with carbohydrate loading; however, 117 ERAS patients had fasting durations ≥6 h, indicating substantial but incomplete overlap between fasting duration and ERAS implementation. Accordingly, the <6 h category was interpreted as an ERAS-associated shortened-fasting strategy incorporating preoperative carbohydrate loading rather than shortened fasting in isolation.

### 2.4. Anesthesia and Perioperative Management

All patients received standardized general anesthesia with endotracheal intubation. Anesthesia was induced with propofol and maintained with either volatile anesthetic agents or total intravenous anesthesia at the discretion of the attending anesthesiologist. Remifentanil was administered intraoperatively as clinically indicated, and neuromuscular blockade was achieved with rocuronium. Standard patient monitoring included noninvasive blood pressure, electrocardiography, and oxygen saturation in all patients. Intraoperative fluid management followed goal-directed therapy principles, with crystalloid solutions used as the primary replacement fluid and colloids or blood products administered as needed according to hemodynamic parameters and estimated blood loss. Collected intraoperative variables included hemodynamic parameters, such as mean blood pressure, heart rate, oxygen saturation, and, when available, mean arterial pressure and central venous pressure; total fluid administration; anesthetic agent consumption; opioid requirements; estimated blood loss; urine output; use of vasopressors or inotropes; and the duration of surgery and anesthesia. Postoperative pain management followed institutional multimodal analgesia protocols consisting of intravenous patient-controlled opioid analgesia, nonopioid analgesics, including acetaminophen and nonsteroidal anti-inflammatory drugs when appropriate, and regional anesthetic techniques, such as transversus abdominis plane blocks, when indicated by the surgical extent and patient characteristics. Patients were initially managed in the post-anesthesia care unit or intensive care unit (ICU), depending on clinical indications. Admission to the ICU was reserved for patients undergoing major hepatobiliary surgery, those with substantial comorbidities, or those requiring prolonged hemodynamic support. Early mobilization and resumption of oral nutrition were encouraged according to ERAS principles.

### 2.5. Data Collection

Demographic data extracted from the electronic medical records included age, sex, body mass index (BMI), and American Society of Anesthesiologists (ASA) physical status classification. Recorded comorbidities included hypertension, diabetes mellitus, cerebrovascular disease, chronic obstructive pulmonary disease, and chronic kidney disease. Surgical variables included the operative organ (liver, bile duct, or pancreas), operative procedure, surgical approach, and operative duration.

Preoperative laboratory variables obtained within 7 days before surgery included CRP, serum albumin, white blood cell count, neutrophil count, lymphocyte count, and absolute lymphocyte count. Postoperative laboratory measurements were extracted for each postoperative day (POD 0–POD 7), when available as part of routine clinical care. Measurements obtained on POD 0, POD 1, POD 3, POD 5, and POD 7 were used for the prespecified longitudinal trajectory analyses and graphical presentation, whereas all available daily measurements from POD 0 through POD 7 were summarized descriptively. Because testing was clinically driven rather than protocol-mandated, measurement availability was recorded at each time point and examined for potential differential missingness between fasting-strategy groups. Characteristics of patients with and without available POD 3 CAR measurements were additionally compared as an assessment of potential informative missingness.

Preoperative fasting duration was calculated as the interval, in minutes, between the last documented caloric intake and the induction of anesthesia. For the categorical analyses, a prespecified 6-h cutoff (<6 h vs. ≥6 h) was used as a pragmatic clinical threshold to distinguish shortened and conventional fasting patterns in our institutional practice. Because fasting duration was defined from the last caloric intake, including carbohydrate-containing beverages, this cutoff should not be interpreted as equivalent to the guideline-recommended 6-h fasting interval for solid food or as a data-derived biological threshold. Information regarding preoperative carbohydrate-containing beverage intake and implementation of the ERAS pathway was also extracted.

### 2.6. Outcome Measures

The primary outcome was the postoperative trajectory of CAR during the first postoperative week. CAR was calculated by dividing serum CRP concentration (mg/dL) by serum albumin concentration (g/dL) at each measured time point. Longitudinal changes in CAR were evaluated using measurements obtained on POD 0, POD 1, POD 3, POD 5, and POD 7.

Secondary outcomes included postoperative trajectories of the NLR and PNI. The NLR was calculated as the ratio of the absolute neutrophil count to the absolute lymphocyte count, whereas the PNI was calculated as 10 × serum albumin (g/dL) + 0.005 × absolute lymphocyte count (cells/μL). Prespecified secondary analyses were performed using POD 3 laboratory measurements to evaluate the associations between preoperative fasting duration, analyzed as a continuous variable, and POD 3 CAR, NLR, and PNI.

Additional clinical outcomes included ICU admission, postoperative hospital length of stay, 30-day readmission, and 30-day mortality.

### 2.7. Statistical Analysis

Continuous variables are presented as mean ± standard deviation or median with interquartile range, as appropriate. Categorical variables are expressed as frequencies and percentages. Patients were categorized according to a prespecified 6-h fasting threshold (<6 h vs. ≥6 h). Patients with missing fasting duration were excluded from analyses involving fasting-group comparisons.

The CAR, NLR, and PNI were calculated at each available postoperative time point. Missing laboratory values were not imputed, and all longitudinal analyses were performed using available data. Measurement availability at each time point was reported as the number and percentage of patients in each fasting-strategy group. Because routine postoperative testing could depend on clinical course, the possibility of informative missingness was additionally explored by comparing baseline and perioperative characteristics between patients with and without available POD 3 CAR measurements.

Between-group comparisons at individual postoperative time points were performed using the Mann–Whitney U test for continuous variables and the chi-square test or Fisher’s exact test for categorical variables, as appropriate.

Postoperative trajectories of CAR, NLR, and PNI were evaluated using linear mixed-effects models with patient-specific random intercepts to account for repeated measurements. Postoperative time was treated as a categorical variable. Fixed effects included fasting group, postoperative time, and fasting group × postoperative time interaction. The significance of the interaction term was used to determine whether postoperative trajectories differed according to preoperative fasting strategy. Observed mean values with standard errors were summarized descriptively, whereas statistical inference was based on the mixed-effects models.

Prespecified secondary analyses were performed using POD 3 laboratory measurements. Because CAR and NLR exhibited right-skewed distributions, both variables were natural log-transformed before multivariable linear regression analyses. Exponentiated regression coefficients are presented as geometric mean ratios with corresponding 95% confidence intervals, representing the relative change in the outcome per 1-h increase in preoperative fasting duration. The primary adjusted models included age and sex. Fully adjusted models additionally included BMI, ASA physical status, operative organ, surgical approach, and operative duration. PNI was analyzed on its original scale, and results are presented as adjusted mean differences per 1-h increase in fasting duration. To address confounding by the ERAS pathway and carbohydrate loading, sensitivity analyses additionally adjusted for ERAS implementation and were repeated after restricting the cohort to ERAS patients. To assess potential nonlinearity, restricted cubic spline models were fitted using four knots at the 5th, 35th, 65th, and 95th percentiles of the fasting-duration distribution (2.00, 3.13, 6.35, and 13.58 h, respectively), with adjustment for age, sex, BMI, ASA physical status, operative organ, surgical approach, and operative duration. Overall associations and nonlinearity were evaluated using nested-model tests.

A two-sided *p* value < 0.05 was considered statistically significant. All statistical analyses were performed using R version 4.2.0 (R Foundation for Statistical Computing, Vienna, Austria).

## 3. Results

### 3.1. Patient Characteristics and Study Cohort

**Figure 1:**
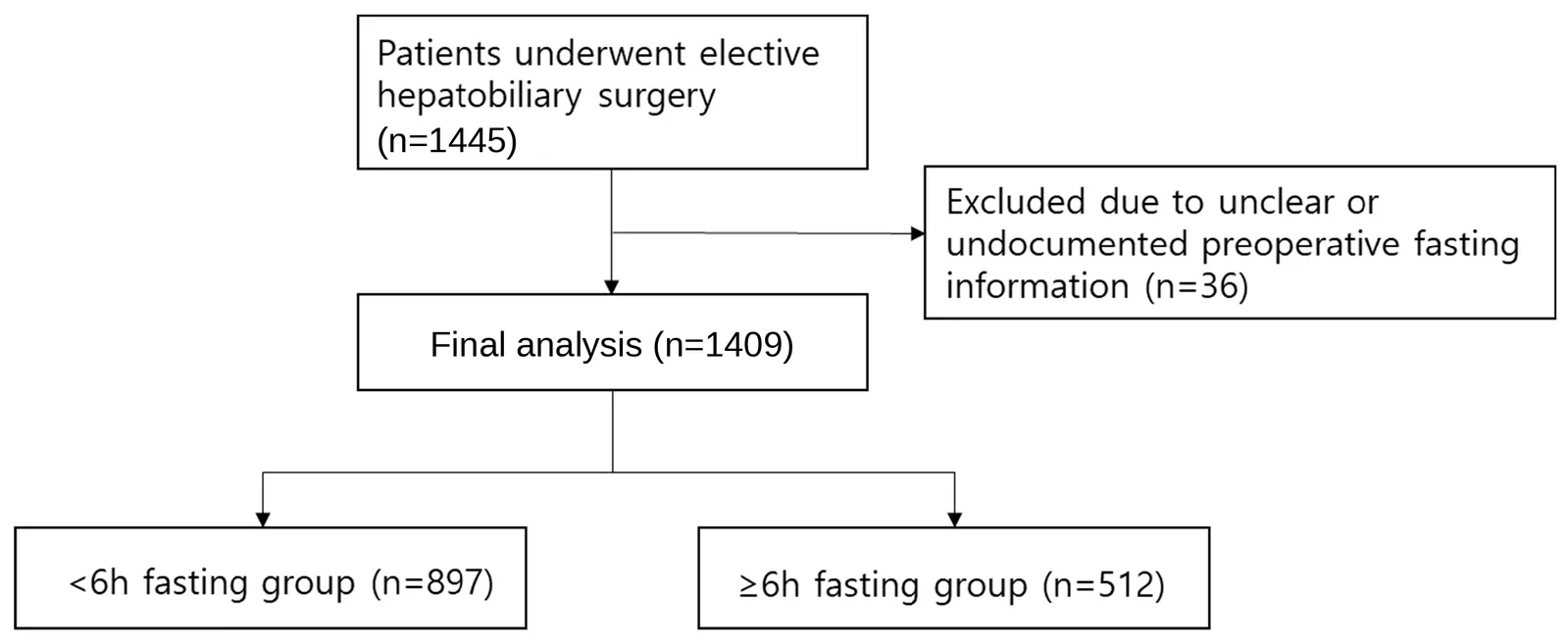
Flowchart of patient selection and study cohort.

A total of 1445 consecutive patients who underwent elective hepatobiliary surgery during the study period were identified. After excluding 36 patients with unclear or undocumented preoperative fasting information, 1409 patients were included in the final analysis. Of these, 897 patients were classified into the <6 h fasting group and 512 into the ≥6 h fasting group (Figure 1). Baseline demographic, clinical, operative, and laboratory characteristics are summarized in Table 1, with no statistically significant differences observed between the two groups.

### 3.2. Primary Outcome: Postoperative Inflammatory–Nutritional Trajectories

**Figure 2:**
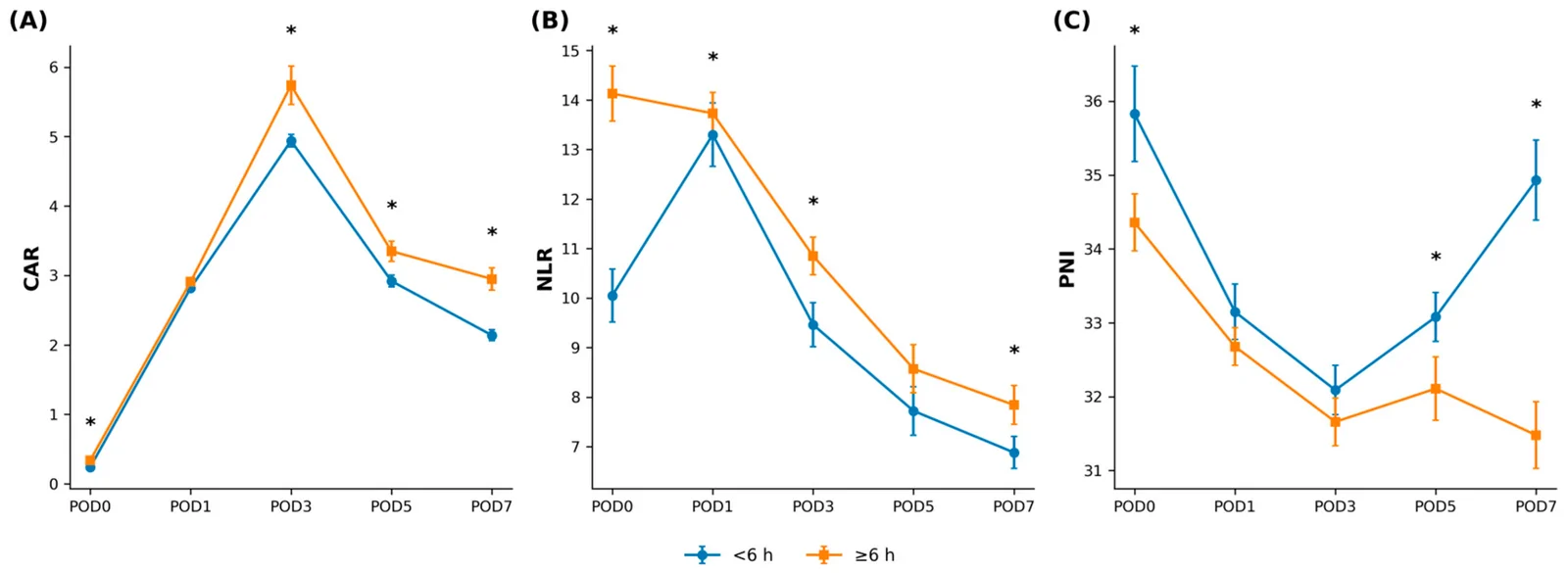
Postoperative inflammatory–nutritional trajectories according to preoperative fasting strategy. Observed mean values with standard errors are shown for the (**A**) *C*-reactive protein-to-albumin ratio (CAR), (**B**) neutrophil-to-lymphocyte ratio (NLR), and (**C**) prognostic nutritional index (PNI) on postoperative days (PODs) 0, 1, 3, 5, and 7 in patients with preoperative fasting durations of <6 h and ≥6 h. The number of patients contributing data at each postoperative time point is shown below the x-axis. Overall differences in temporal trajectories were evaluated using linear mixed-effects models including fasting group, postoperative time, and the fasting group × postoperative time interaction as fixed effects and a patient-specific random intercept. Error bars represent standard errors. Asterisks (*) indicate time points at which the between-group difference was statistically significant (*p* < 0.05).

Postoperative inflammatory and nutritional indices according to preoperative fasting strategy are summarized in Table 2, and their temporal trajectories are illustrated in Figure 2. Because postoperative laboratory testing was performed according to clinical indications, measurement availability varied substantially across biomarkers, postoperative time points, and fasting-strategy groups. The number and percentage of patients contributing measurements at each time point are therefore reported in Table 2. In an additional comparison of patients with and without available POD 3 CAR measurements, availability was associated with age, ASA physical status, fasting strategy, and ERAS implementation, whereas BMI, sex, and operative duration were similar (Supplementary Table S1). These findings indicate that informative missingness cannot be excluded.

The CAR increased markedly during the early postoperative period in both groups and gradually declined thereafter. Compared with patients who fasted for <6 h, those who fasted for ≥6 h had significantly higher CAR values on POD 0, POD 2, POD 3, POD 4, POD 5, POD 6, and POD 7, whereas no significant between-group difference was observed on POD 1 (Table 2). In the linear mixed-effects model, the fasting group × postoperative time interaction was significant (*p* < 0.001), indicating that postoperative CAR trajectories differed according to preoperative fasting strategy (Figure 2A). Significant overall effects of fasting group and postoperative time were also observed (both *p* < 0.001).

The NLR showed a similar postoperative pattern, characterized by an early increase followed by gradual recovery. Patients in the ≥6 h fasting group had significantly higher NLR values on POD 0, POD 1, POD 2, POD 3, POD 5, and POD 7, whereas no significant differences were observed on POD 4 or POD 6 (Table 2). The fasting group × postoperative time interaction was significant in the linear mixed-effects model (*p* < 0.001), confirming that postoperative NLR trajectories differed between the two fasting groups (Figure 2B). Significant overall effects of fasting group and postoperative time were also observed (both *p* < 0.001).

The PNI decreased immediately after surgery and gradually recovered during the postoperative period. Compared with the <6 h fasting group, the ≥6 h fasting group had significantly lower PNI values on POD 0, POD 5, POD 6, and POD 7, whereas no significant differences were observed from POD 1 through POD 4 (Table 2). Linear mixed-effects analysis demonstrated a significant fasting group × postoperative time interaction (*p* = 0.003), indicating that postoperative PNI trajectories differed according to preoperative fasting strategy (Figure 2C). Significant overall effects of fasting group (*p* = 0.002) and postoperative time (*p* < 0.001) were also observed.

### 3.3. Secondary Outcome: Clinical Outcomes

Clinical outcomes are summarized in Table 3. ICU admission occurred in 106 (11.8%) patients in the <6 h fasting group and 54 (10.5%) patients in the ≥6 h fasting group (*p* = 0.470). The mean postoperative hospital stay was 14.8 ± 8.2 days and 14.2 ± 7.5 days, respectively (*p* = 0.412). Thirty-day readmission occurred in 39 (4.3%) and 20 (3.9%) patients (*p* = 0.691), whereas 30-day mortality occurred in 4 (0.4%) patients in the <6 h fasting group and in no patients in the ≥6 h fasting group (*p* = 1.000, Fisher’s exact test). None of these clinical outcomes differed significantly between the groups.

### 3.4. Secondary Outcome: Associations Between Fasting Duration and POD 3 Inflammatory–Nutritional Indices

In prespecified analyses treating preoperative fasting duration as a continuous variable, longer fasting duration remained significantly associated with higher POD 3 CAR (geometric mean ratio, 1.012; 95% CI, 1.004–1.021; *p* = 0.005) and NLR (geometric mean ratio, 1.031; 95% CI, 1.017–1.046; *p* < 0.001). The association with POD 3 PNI remained non-significant (adjusted mean difference, −0.053; 95% CI, −0.164 to 0.057; *p* = 0.345).

The associations were substantially attenuated after additional adjustment for ERAS implementation. Fasting duration was no longer significantly associated with POD 3 CAR (geometric mean ratio, 1.007; 95% CI, 0.994–1.021; *p* = 0.302) or NLR (geometric mean ratio, 1.023; 95% CI, 1.000–1.047; *p* = 0.053). In contrast, an inverse association between fasting duration and POD 3 PNI emerged after additional adjustment for ERAS implementation (adjusted mean difference, −0.219; 95% CI, −0.397 to −0.041; *p* = 0.016). In analyses restricted to ERAS patients, fasting duration was likewise not associated with CAR (geometric mean ratio, 1.003; 95% CI, 0.988–1.018; *p* = 0.724) or NLR (geometric mean ratio, 1.014; 95% CI, 0.983–1.046; *p* = 0.390), whereas the inverse association with PNI remained statistically significant (adjusted mean difference, −0.230; 95% CI, −0.456 to −0.004; *p* = 0.048). These sensitivity analyses suggest that the primary associations cannot be attributed to fasting duration alone and may partly reflect the broader ERAS-associated strategy incorporating carbohydrate loading.

**Figure 3:**
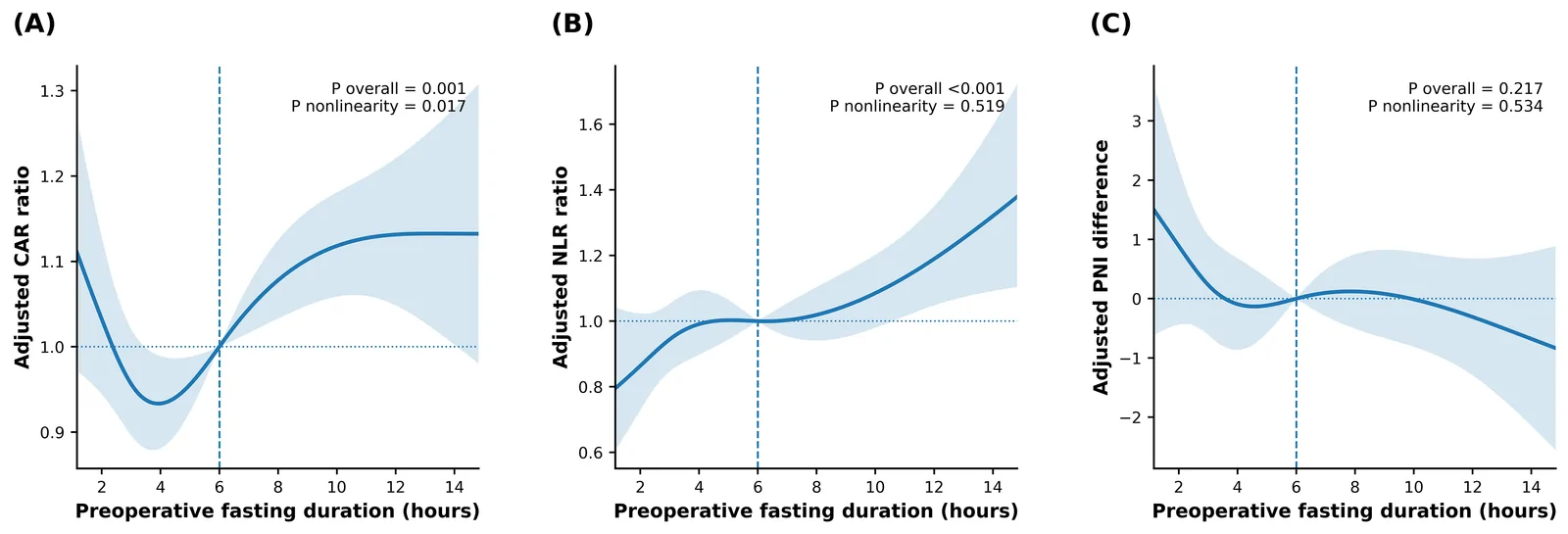
Restricted cubic spline associations between preoperative fasting duration and inflammatory–nutritional indices on postoperative day 3. Adjusted restricted cubic spline curves show the associations between preoperative fasting duration and (**A**) *C*-reactive protein-to-albumin ratio (CAR), (**B**) neutrophil-to-lymphocyte ratio (NLR), and (**C**) prognostic nutritional index (PNI) on postoperative day (POD) 3. Models were adjusted for age, sex, body mass index, ASA physical status, operative organ, surgical approach, and operative duration. Four knots were placed at the 5th, 35th, 65th, and 95th percentiles of fasting duration. CAR and NLR are presented as adjusted geometric mean ratios and PNI as an adjusted mean difference, with 6 h used as the reference value. Solid curves represent adjusted estimates and shaded areas represent 95% confidence intervals. The vertical dashed line at 6 h indicates the prespecified pragmatic cutoff used for categorical analyses and does not represent a data-derived threshold.

Restricted cubic spline analyses further characterized the continuous associations between fasting duration and POD 3 biomarkers (Figure 3). For CAR, the overall association was significant (*p* = 0.001), with evidence of nonlinearity (*p* for nonlinearity = 0.017). For NLR, the overall association was significant (*p* < 0.001), with no evidence of nonlinearity (*p* for nonlinearity = 0.519). No significant overall or nonlinear association was observed for PNI (*p* = 0.217 and *p* for nonlinearity = 0.534, respectively). The spline analyses did not identify 6 h as a discrete biological threshold. Regression and sensitivity analyses are summarized in Table 3B.

## 4. Discussion

In this large retrospective cohort study of patients undergoing elective hepatobiliary surgery, patients managed with a shortened-fasting strategy showed more favorable postoperative inflammatory–nutritional profiles, particularly for CAR and NLR, than those managed with a longer-fasting strategy. Significant differences in the postoperative trajectories of CAR and NLR were observed between the two fasting-strategy groups, and continuous analyses further demonstrated associations of longer fasting duration with higher POD 3 CAR and NLR. These findings support an association between perioperative fasting strategy and postoperative inflammatory–nutritional responses. However, because shortened fasting in our cohort was closely integrated with ERAS implementation and preoperative carbohydrate loading, the observed differences cannot be attributed to fasting duration alone. The attenuation of the CAR and NLR associations after adjustment for ERAS implementation and in ERAS-only analyses suggests that the broader ERAS-associated perioperative strategy contributed importantly to the observed relationships.

Among the biomarkers evaluated, CAR demonstrated the most consistent between-group discrimination across the postoperative period. CAR integrates two complementary biological processes: the acute inflammatory response reflected by CRP and the nutritional and hepatic synthetic function reflected by serum albumin. Compared with either biomarker alone, CAR provides a more comprehensive assessment of postoperative inflammatory–nutritional balance and has been associated with postoperative morbidity, prolonged hospitalization, and adverse long-term outcomes across various surgical populations, including patients undergoing hepatobiliary surgery [4,6,8,19,20,21,22,23]. Our findings extend these observations by demonstrating distinct postoperative CAR trajectories according to perioperative fasting strategy. The association observed in the primary analyses was attenuated after accounting for ERAS implementation, suggesting that the favorable CAR profile associated with the shortened-fasting strategy may reflect the combined effects of multiple perioperative interventions rather than fasting duration in isolation.

The observed findings are biologically plausible in the context of contemporary perioperative care. Major hepatobiliary surgery elicits substantial metabolic and inflammatory stress, while prolonged fasting may exacerbate glycogen depletion, insulin resistance, and catabolic hormonal responses [2,3,24]. ERAS pathways are designed to mitigate these effects by avoiding unnecessary fasting and providing preoperative carbohydrate-containing beverages [14,15,17,18,25,26,27,28]. Carbohydrate loading may provide metabolic effects beyond shortening the fasting interval itself, including stimulation of endogenous insulin secretion and attenuation of postoperative insulin resistance [14,15,17,18,25,26,27,28,29,30,31]. Thus, the more favorable CAR and NLR trajectories observed in the shortened-fasting group are consistent with the potential metabolic benefits of contemporary ERAS-associated perioperative management. However, because shortened fasting, carbohydrate loading, and ERAS implementation were closely linked in this cohort, their individual contributions to the observed biomarker differences cannot be determined separately.

The NLR demonstrated postoperative changes broadly similar to those observed for CAR, further supporting an association between fasting strategy and the early postoperative inflammatory response. In contrast, PNI showed a less consistent pattern. Although the overall PNI trajectory differed significantly between fasting-strategy groups, between-group differences were not significant from POD 1 through POD 4, and fasting duration was not significantly associated with POD 3 PNI in the primary fully adjusted continuous analysis. An inverse association with PNI emerged after adjustment for ERAS implementation and remained significant in the ERAS-only analysis. Given that this association was not observed in the primary fully adjusted or restricted cubic spline analyses, the PNI findings should be interpreted more cautiously than those for CAR and NLR. PNI reflects nutritional reserve in addition to systemic inflammation and may be influenced by hepatic synthetic function, nutritional support, fluid balance, and other perioperative factors [9,11,32]. Accordingly, PNI may capture aspects of postoperative recovery that differ from those reflected by CAR and NLR.

The continuous and restricted cubic spline analyses provided additional context for the primary categorical findings. Longer fasting duration was associated with higher POD 3 CAR and NLR in the primary fully adjusted continuous models, supporting the differences observed between fasting-strategy groups. Restricted cubic spline analyses further suggested a nonlinear association for CAR and an approximately linear association for NLR. However, the spline analyses did not identify 6 h as a discrete biological threshold. Accordingly, the 6-h cutoff used in the present study should be regarded as a pragmatic categorization reflecting the perioperative fasting strategies used in clinical practice rather than as a physiological threshold. Furthermore, the attenuation of the continuous CAR and NLR associations after accounting for ERAS implementation indicates that fasting duration alone does not fully explain the observed differences between fasting-strategy groups.

This study has several strengths. It included a large cohort of patients undergoing hepatobiliary surgery and comprehensively evaluated complementary inflammatory and nutritional biomarkers throughout the first postoperative week. Longitudinal biomarker trajectories were analyzed using linear mixed-effects models, while fasting duration was additionally evaluated as a continuous exposure using restricted cubic spline analyses. These complementary approaches allowed us to characterize both differences according to clinically relevant fasting strategy and the relationship between actual fasting duration and postoperative biomarkers. In addition, ERAS-adjusted sensitivity analysis allowed us to assess the extent to which the observed fasting-duration associations were influenced by the close relationship among shortened fasting, ERAS implementation, and carbohydrate loading.

These findings are consistent with contemporary perioperative recommendations that discourage unnecessarily prolonged fasting and permit carbohydrate-containing beverages before surgery in appropriately selected patients [5,26,29,31,33]. The present findings suggest that a perioperative strategy incorporating shortened fasting within an ERAS pathway is associated with more favorable postoperative CAR and NLR profiles. Nevertheless, they should not be interpreted as demonstrating that shortening fasting duration alone is responsible for these differences. Rather, the results support the potential value of the integrated perioperative strategy, including shortened fasting and carbohydrate loading, while highlighting the need to determine the relative contribution of each component in future studies.

Despite the differences in postoperative biomarker profiles, no significant differences were observed in ICU admission, postoperative hospital stay, 30-day readmission, or 30-day mortality. This may reflect the relatively low event rates for these clinical outcomes or indicate that differences in inflammatory–nutritional biomarkers precede clinically detectable differences in recovery. Moreover, established minimal clinically important differences for postoperative CAR, NLR, or PNI trajectories are lacking. Therefore, the statistically significant biomarker differences observed in this study cannot be assumed to represent clinically meaningful improvements in recovery and should primarily be regarded as physiological findings that warrant further prospective investigation.

Several limitations should be acknowledged. First, the retrospective observational design precluded causal inference, and residual confounding cannot be completely excluded despite multivariable adjustment. Second, postoperative laboratory measurements were obtained as part of routine clinical care rather than a standardized study protocol, resulting in substantial variation in measurement availability across biomarkers, time points, and fasting-strategy groups. Although linear mixed-effects models accommodate unbalanced repeated measurements, bias may persist when measurement availability depends on postoperative clinical condition. The observed differences between patients with and without available POD 3 CAR measurements and the low measurement rates at some later time points indicate that informative missingness cannot be excluded; therefore, later longitudinal estimates should be interpreted cautiously. Third, shortened fasting was closely linked to ERAS implementation and preoperative carbohydrate loading. Although ERAS-adjusted and ERAS-only sensitivity analyses were performed, the independent contributions of fasting duration, carbohydrate loading, and other ERAS components could not be fully disentangled in this observational study. Finally, this was a single-center study, and validation in prospective multicenter studies is warranted.

## 5. Conclusions

An ERAS-associated perioperative strategy incorporating shortened preoperative fasting and carbohydrate loading was associated with more favorable postoperative CAR and NLR trajectories, whereas PNI findings were less consistent. However, these associations were influenced by ERAS implementation and should not be attributed to fasting duration alone. Prospective studies are needed to clarify the independent contributions of fasting duration, carbohydrate loading, and other ERAS components.

## Figures and Tables

**Table 1:** Baseline demographic, clinical, operative, and preoperative laboratory characteristics according to preoperative fasting strategy.

Characteristic	<6 h (*n* = 897)	≥6 h (*n* = 512)	*p* Value
Age, years	63.5 ± 9.3	64.0 ± 10.6	0.576
Male sex, *n* (%)	551 (61.4%)	314 (61.3%)	0.925
BMI, kg/m^2^	24.2 ± 3.2	23.8 ± 3.2	0.138
ASA physical status, *n*			
I/II/III	73/699/125	37/411/64	
Comorbidities			
Hypertension, *n* (%)	356 (39.7%)	205 (40.0%)	0.932
Diabetes mellitus, *n* (%)	234 (26.1%)	141 (27.5%)	0.553
Cerebrovascular disease, *n* (%)	26 (2.9%)	10 (2.0%)	0.279
Chronic kidney disease, *n* (%)	16 (1.8%)	7 (1.4%)	0.749
Operative organ			0.954
Pancreas	647 (72.1%)	373 (72.9%)	
Liver and others	250 (27.9%)	139 (27.1%)	
Surgical approach			0.682
Open	655 (73.0%)	379 (74.0%)	
Laparoscopic/robotic	242 (27.0%)	133 (26.0%)	
Anesthesia type			0.882
Inhalation	874 (97.4%)	497 (97.1%)	
TIVA	23 (2.6%)	15 (2.9%)	
Operative duration, min	263 [226, 309]	252.00 [214, 307]	0.325
Anesthesia duration, min	326 [287, 365]	313 [265.50, 369]	0.342
Preoperative laboratory findings			
CRP, mg/dL	0.82 ± 1.95	0.74 ± 1.78	0.639
Albumin, g/dL	3.50 ± 0.46	3.44 ± 0.45	0.150
White blood cell count, ×10^3^/μL	6.5 [5.2, 8.2]	6.8 [5.5, 8.5]	0.125
Neutrophil count, ×10^3^/μL	62.4 ± 10.5	61.2 ± 9.8	0.185
Lymphocyte count, ×10^3^/μL	28.5 [21.2, 35.8]	30.1 [22.5, 36.5]	0.102
Absolute lymphocyte count, cells/μL	1760 [1350, 2150]	1850 [1440, 2265]	0.113

Data are presented as mean ± standard deviation, median [interquartile range], or number (%), as appropriate. BMI, body mass index; ASA, American Society of Anesthesiologists; CRP, *C*-reactive protein; TIVA, total intravenous anesthesia.

**Table 2:** Postoperative inflammatory and nutritional indices according to preoperative fasting strategy.

Time	CAR (<6 h)	CAR (≥6 h)	*p* Value	NLR (<6 h)	NLR (≥6 h)	*p* Value	PNI (<6 h)	PNI (≥6 h)	*p* Value
POD 0	0.24 ± 0.51 (*n* = 664; 74.0%)	0.34 ± 0.73 (*n* = 423; 82.6%)	<0.001	10.05 ± 8.58 (*n* = 258; 28.8%)	14.13 ± 10.14 (*n* = 335; 65.4%)	<0.001	35.83 ± 9.56 (*n* = 220; 24.5%)	34.36 ± 6.92 (*n* = 324; 63.3%)	0.002
POD 1	2.82 ± 1.34 (*n* = 869; 96.9%)	2.91 ± 1.39 (*n* = 506; 98.8%)	0.374	13.30 ± 9.72 (*n* = 229; 25.5%)	13.73 ± 7.63 (*n* = 330; 64.5%)	0.013	33.15 ± 5.64 (*n* = 226; 25.2%)	32.68 ± 4.64 (*n* = 338; 66.0%)	0.178
POD 2	5.82 ± 2.36 (*n* = 866; 96.5%)	6.17 ± 2.18 (*n* = 503; 98.2%)	<0.001	11.60 ± 7.48 (*n* = 183; 20.4%)	12.41 ± 5.88 (*n* = 294; 57.4%)	0.009	32.64 ± 4.08 (*n* = 188; 21.0%)	32.26 ± 4.06 (*n* = 307; 60.0%)	0.498
POD 3	4.94 ± 2.43 (*n* = 734; 81.8%)	5.74 ± 5.01 (*n* = 331; 64.6%)	<0.001	9.46 ± 5.71 (*n* = 164; 18.3%)	10.85 ± 4.77 (*n* = 155; 30.3%)	0.001	32.09 ± 4.28 (*n* = 164; 18.3%)	31.66 ± 4.02 (*n* = 154; 30.1%)	0.193
POD 4	3.76 ± 2.27 (*n* = 459; 51.2%)	4.35 ± 2.45 (*n* = 379; 74.0%)	<0.001	7.90 ± 4.58 (*n* = 224; 25.0%)	7.82 ± 4.75 (*n* = 276; 53.9%)	0.585	32.26 ± 4.37 (*n* = 204; 22.7%)	31.64 ± 4.10 (*n* = 301; 58.8%)	0.138
POD 5	2.92 ± 2.15 (*n* = 679; 75.7%)	3.35 ± 2.40 (*n* = 268; 52.3%)	0.015	7.72 ± 6.92 (*n* = 200; 22.3%)	8.57 ± 5.90 (*n* = 147; 28.7%)	0.011	33.08 ± 4.67 (*n* = 201; 22.4%)	32.11 ± 5.09 (*n* = 141; 27.5%)	0.043
POD 6	2.48 ± 2.03 (*n* = 395; 44.0%)	3.17 ± 2.33 (*n* = 356; 69.5%)	<0.001	6.97 ± 4.48 (*n* = 202; 22.5%)	7.25 ± 4.56 (*n* = 262; 51.2%)	0.254	34.20 ± 4.83 (*n* = 188; 21.0%)	32.46 ± 4.27 (*n* = 307; 60.0%)	<0.001
POD 7	2.14 ± 1.98 (*n* = 628; 70.0%)	2.95 ± 2.59 (*n* = 260; 50.8%)	<0.001	6.88 ± 4.83 (*n* = 223; 24.9%)	7.84 ± 4.79 (*n* = 150; 29.3%)	0.004	34.93 ± 4.06 (*n* = 56; 6.2%)	31.48 ± 4.33 (*n* = 92; 18.0%)	<0.001

Data are presented as mean ± standard deviation. The sample size at each postoperative time point is shown as n (percentage of the corresponding fasting-strategy group) and reflects patients with available laboratory measurements. *p* values were calculated using the Mann–Whitney U test. CAR, CRP-to-albumin ratio; NLR, neutrophil-to-lymphocyte ratio; PNI, prognostic nutritional index; POD, postoperative day.

**Table 3:** Secondary clinical outcomes and associations between preoperative fasting duration and postoperative inflammatory–nutritional indices. (**A**) Clinical outcomes; (**B**) Associations between preoperative fasting duration and inflammatory–nutritional indices on postoperative day 3.

(**A**)				
		<6 h (*n* = 897)	≥6 h (*n* = 512)	*p* value
ICU admission, *n* (%)		106 (11.8%)	54 (10.5%)	0.470
Postoperative hospital stay, days		14.8 ± 8.2	14.2 ± 7.5	0.412
30-day readmission, *n* (%)		39 (4.3%)	20 (3.9%)	0.691
30-day mortality, *n* (%)		4 (0.4%)	0 (0.0%)	1.000
(**B**)				
	Model	Estimate	95% CI	*p* value
CAR	Unadjusted	1.014	1.005–1.023	0.002
CAR	Age- and sex-adjusted	1.014	1.005–1.023	0.002
CAR	Fully adjusted	1.012	1.004–1.021	0.005
CAR	Fully adjusted + ERAS	1.007	0.994–1.021	0.302
CAR	ERAS-only fully adjusted	1.003	0.988–1.018	0.724
NLR	Unadjusted	1.029	1.016–1.043	<0.001
NLR	Age- and sex-adjusted	1.029	1.016–1.042	<0.001
NLR	Fully adjusted	1.031	1.017–1.046	<0.001
NLR	Fully adjusted + ERAS	1.023	1.000–1.047	0.053
NLR	ERAS-only fully adjusted	1.014	0.983–1.046	0.390
PNI	Unadjusted	−0.087	−0.190 to 0.016	0.097
PNI	Age- and sex-adjusted	−0.089	−0.192 to 0.015	0.093
PNI	Fully adjusted	−0.053	−0.164 to 0.057	0.345
PNI	Fully adjusted + ERAS	−0.219	−0.397 to −0.041	0.016
PNI	ERAS-only fully adjusted	−0.230	−0.456 to −0.004	0.048

ICU, intensive care unit. Data are presented as geometric mean ratios (95% CIs) per 1-h increase in preoperative fasting duration for CAR and NLR and as mean differences (95% CIs) for PNI. The age- and sex-adjusted model included age and sex. The fully adjusted model additionally included BMI, ASA physical status, operative organ, surgical approach, and operative duration. The fully adjusted + ERAS model additionally included ERAS implementation. ERAS-only analyses were restricted to patients managed under the ERAS pathway and used the same fully adjusted covariates. CAR, *C*-reactive protein-to-albumin ratio; NLR, neutrophil-to-lymphocyte ratio; PNI, prognostic nutritional index; CI, confidence interval; ASA, American Society of Anesthesiologists.

## Data Availability

The data that support the findings of this study are available from the corresponding author upon reasonable request.

## Supplementary Materials

The following supporting information can be downloaded at: https://www.mdpi.com/article/10.3390/nu18172922/s1, Table S1: Characteristics of patients with and without available POD 3 CAR measurements.

## Abbreviations

The following abbreviations are used in this manuscript:

ASA	American Society of Anesthesiologists
BMI	Body mass index
CAR	*C*-reactive protein-to-albumin ratio
CI	Confidence interval
CRP	*C*-reactive protein
ERAS	Enhanced Recovery After Surgery
ICU	Intensive care unit
IQR	Interquartile range
NLR	Neutrophil-to-lymphocyte ratio
POD	Postoperative day
PNI	Prognostic nutritional index
SD	Standard deviation
STROBE	Strengthening the Reporting of Observational Studies in Epidemiology
TIVA	Total intravenous anesthesia
