# Supplementary material for: Association of Preoperative Fasting Strategy with Postoperative Inflammatory–Nutritional Trajectories and Clinical Outcomes Following Hepatobiliary Surgery: A Retrospective Cohort Study
Source: Nutrients. 2026 Sep 7;18(17):2922. doi: 10.3390/nu18172922 (PMC13567360; doi:10.3390/nu18172922)
Supplement: Supplementary file 1 [file nutrients-18-02922-s001.zip › nutrients-4502952-supplementary.pdf]

Supplementary Table S1. Characteristics of patients with and without available POD 3 CAR measurements.

Characteristic	POD 3 CAR available (n = 1,065)	POD 3 CAR unavailable (n = 344)	P value
Age, years	64.9 ± 9.7	63.0 ± 9.7	0.002
Male sex, n (%)	679 (63.8%)	203 (59.0%)	0.129
BMI, kg/m^2	23.7 ± 3.4	23.7 ± 3.3	0.863
ASA physical status, I/II/III/IV	41/882/141/1	30/266/48/0	0.003
Operative duration, min	257.0 [205.0, 307.0]	252.5 [209.0, 303.0]	0.681
Fasting duration, h	3.8 [2.5, 8.0]	8.0 [3.1, 11.2]	<0.001
<6 h fasting, n (%)	734 (68.9%)	163 (47.4%)	<0.001
ERAS pathway, n (%)	842 (79.1%)	172 (50.0%)	<0.001

Data are presented as mean ± standard deviation, median [interquartile range], or number (%), as appropriate. This analysis was performed to assess potential informative missingness at the prespecified POD 3 time point. CAR, C-reactive protein-to-albumin ratio; BMI, body mass index; ASA, American Society of Anesthesiologists; ERAS, Enhanced Recovery After Surgery; POD, postoperative day.
